# Genetic Variation and Divergence of Genes Involved in Leaf Adaxial-Abaxial Polarity Establishment in *Brassica rapa*

**DOI:** 10.3389/fpls.2016.00094

**Published:** 2016-02-09

**Authors:** Jianli Liang, Bo Liu, Jian Wu, Feng Cheng, Xiaowu Wang

**Affiliations:** Molecular Genetics Lab, Biotechnology Department, Institute of Vegetables and Flowers, Chinese Academy of Agricultural SciencesBeijing, China

**Keywords:** Chinese cabbage, *Brassica rapa*, adaxial-abaxial polarity, genetic variation, purifying selection, leafy head

## Abstract

Alterations in leaf adaxial-abaxial (ad-ab) polarity are one of the main factors that influence leaf curvature. In Chinese cabbage, leaf incurvature is an essential prerequisite to the formation of a leafy head. Identifying ad-ab patterning genes and investigating their genetic variation may facilitate elucidation of the mechanisms underlying leaf incurvature during head formation. Comparative genomic analysis of 45 leaf ad-ab patterning genes in *Brassica rapa* based on 26 homologs of *Arabidopsis thaliana* indicated that these genes underwent expansion and were retained after whole genome triplication (WGT). We also assessed the nucleotide diversity and selection footprints of these 45 genes in a collection of 94 *Brassica rapa* accessions that were composed of heading and non-heading morphotypes. Six of the 45 genes showed significant negative Tajima's D indices and nucleotide diversity reduction in heading accessions compared to those in non-heading accessions, indicating that they underwent purifying selection. Further testing of the *BrARF3.1* gene, which was one of the selection signals from a larger collection, confirmed that purifying selection did occur. Our results provide genetic evidence that ad-ab patterning genes are involved in leaf incurvature, which is associated with formation of a leafy head, as well as promote an understanding of the genetic mechanism underlying leafy head formation in Chinese cabbage.

## Introduction

Chinese cabbage (*Brassica rapa* L. ssp. *pekinensis*) is widely cultivated and one of the most consumed vegetable crops in Asia. It has a uniform, compact leafy head composed of several enlarged, winged, and crinkly incurved leaves that have been utilized as highly nutritious food. Because head traits such as size, shape, and weight contribute to yield and quality, these features are considered to be of high commercial value (Yu et al., 2013; Mao et al., 2014).

**Figure 1:**
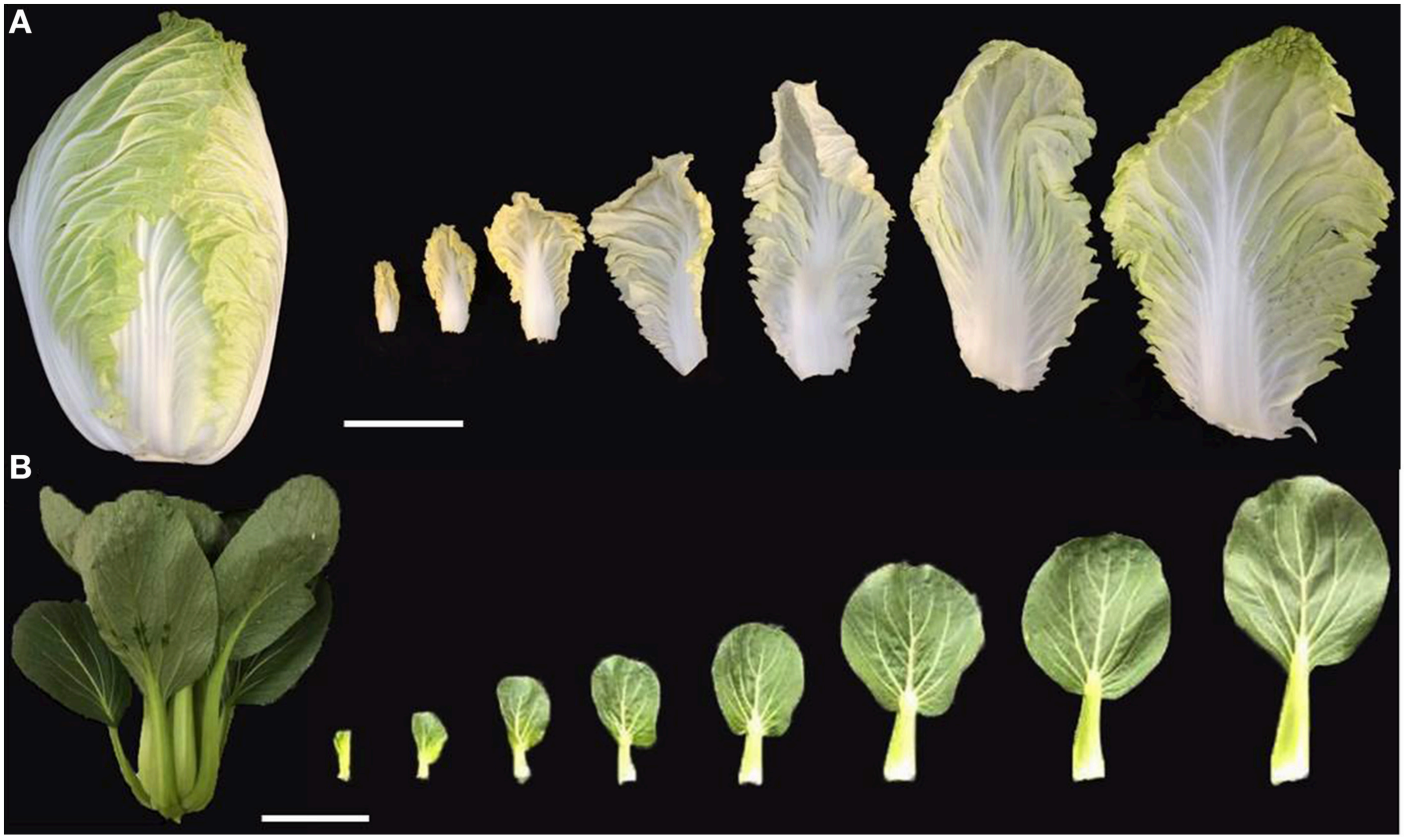
**Leaf morphology of heading and non-heading *B. rapa* in mature stage**. **(A)** The leafy head of heading *B. rapa* (Chinese cabbage), and its incurved leaves from inside to outside. **(B)** The non-heading *B. rapa* (pak choi) with flat leaves from inside to outside. Scale bars: 10 cm.

To produce a leafy head, Chinese cabbage undergoes three developmental stages, namely, seedling, rosette, and heading (Yu et al., 2013). The seedling leaves and rosette leaves are flat, whereas the head leaves within the leafy head show incurvature with a larger abaxial (bottom) surface. In addition, compared to other non-heading *B. rapa*, Chinese cabbage also shows significant differences in leaf morphology such as leaf shape, size, bulges, lobes, petioles, and blade base angles (Figure 1), indicating that leaf heading is a complex quantitative trait influenced by multiple genes (Yu et al., 2013; Wang et al., 2014). Quantitative trait locus (QTL) mapping of leafy heads using a recombinant inbred line (RIL) population previously identified three candidate genes, *BrpGL1, BrpESR1*, and *BrpSAW1*, which regulate development of trichome, petiole, serration, and cell division and possibly contribute to the formation of leafy heads (Yu et al., 2013). The global analysis of the transcriptomes from rosette leaves and folding leaves in Chinese cabbage using RNA-sequencing (RNA-Seq) revealed that the regulation of transcription factors, protein kinases and calcium may play critical roles in controlling leafy head development besides some stimuli, such as carbohydrate levels, light intensity and endogenous hormones (Wang et al., 2012). In addition, because leaf incurvature is an essential prerequisite to leafy head formation (Mao et al., 2014), genetic studies involving leaf incurvature may lead to a better understanding of the molecular mechanism underlying leafy head formation in Chinese cabbage.

Most plant species have evolved flat leaves that further differentiate into two specialized leaf domains, namely, the adaxial (top) and abaxial surfaces. The adaxial side of the leaf originates closest to the meristem and develops a tightly packed layer of palisade mesophyll cells that facilitate in the capture of light, whereas the abaxial side contains a loosely packed spongy mesophyll layer and a higher density of stomatal pores that assist in gas exchange and regulate transpiration (Braybrook and Kuhlemeier, 2010; Moon and Hake, 2011; Yamaguchi et al., 2012). The developmental mechanism behind leaf ad-ab polarity specification and flat lamina formation has always been of interest to biologists. Several regulators involved in leaf adaxial-abaxial (ad-ab) polarity specification have now been identified. Further studies have also demonstrated that mutual antagonistic interactions between adaxial and abaxial determinants are not only responsible for ad-ab polarity specification, but also define the boundary between the ad-ab domain, in which subsequent flat lamina growth is promoted (Yamaguchi et al., 2012). Loss- or gain-of-function mutations in adaxial or abaxial polarity genes generally result in loss of lamina flattening and an upward- or downward-curling leaf phenotype due to alterations in the ad-ab patterning system (Kidner and Timmermans, 2007, 2010; Yamaguchi et al., 2012).

The inward-curled rosette leaf phenotype of *Arabidopsis* abaxial polarity mutants exactly resembles the head leaf incurvature underlying the leafy head formation in Chinese cabbage. The *HYL1* gene is responsible for the leaf abaxial determinant-miR166 (Han et al., 2004; Wu et al., 2007), and the rosette leaves of *Arabidopsis hyl1* mutants are curved inward (Wu et al., 2004; Liu et al., 2011). *BcpLH*, which is the homolog of the *Arabidopsis HYL1* gene that was isolated from Chinese cabbage, has been shown to affect the inward curvature of folding leaves (Yu et al., 2000). Moreover, unlike flat seedling leaves and rosette leaves that mainly function in photosynthesis and respiration, head leaves generally serve as storage organs. These findings prompted us to hypothesize that the incurvature of head leaves is associated with alterations in the leaf ad-ab patterning system.

Leaf ad-ab patterning is regulated by several distinct transcription factor (TF) families, of which some are regulated by two kinds of small RNAs, namely, microRNAs (miR165/166) and *trans*-acting short interfering RNAs (*TAS3*-derived tasiR-ARF). In angiosperm models, adaxial polarity is specified by genes of the class III homeodomain-leucine zipper (HD-ZIPIII) family (*REV, PHB, PHV*, and *ATHB8*) (McConnell et al., 2001; Emery et al., 2003), Myb and LOB domain transcription factors *ASYMMETRIC LEAVES1* (*AS1*) and *AS2* (Lin et al., 2003; Iwakawa et al., 2007), and *TAS3*-derived tasiR-ARF (Hunter et al., 2006; Chitwood et al., 2007, 2009). On the other hand, *KANADI* family genes (*KAN1–3*) (Eshed et al., 2001, 2004; Kerstetter et al., 2001), *AUXIN RESPONSE FACTORS* (*ARF*) *ETTIN* (*ETT/ARF3*) and *ARF4* (Pekker et al., 2005), and miR165/166 small RNAs (Kidner and Martienssen, 2004) are abaxial-specific. *YABBY* family genes (*YAB1–3*) were first proposed as abaxial determinants (Stahle et al., 2009). However, recent studies have shown that *YABBY* genes and *WUSCHEL-RELATED HOMEOBOX* genes *(WOX1* and *WOX3)* are middle domain-specific and involved not only in adaxial/abaxial patterning and but also in subsequent flat lamina growth (Kidner and Timmermans, 2007; Vandenbussche et al., 2009; Nakata et al., 2012). The production of microRNAs and tasiRNAs requires a number of proteins, which include AGO1, AGO7, SGS3, RDR6, DCL1, DCL4, and SE (reviewed by Moon and Hake, 2011). The regulatory network controlling ad-ab polarity is based on the mutual antagonistic interaction between adaxial and abaxial determinants. The *KAN* genes are key regulators of abaxial polarity of leaves, whereas HD-ZIPIII genes are major adaxial determinants. KAN and HD-ZIPIII are thought to act antagonistically (Eshed et al., 2004; Huang et al., 2014), whereas AS2 and KAN negatively regulate each other (Lin et al., 2003; Wu et al., 2008). In addition, HD-ZIPIII genes are the targets of miR165/166-mediated gene regulation, and its expression is repressed at the adaxial side. Similar to the HD-ZIPIII genes, the expression of *ARF3/4* abaxial genes is regulated by tasiR-ARF (Fahlgren et al., 2006; Kidner and Timmermans, 2010).

Chinese cabbage is a subspecies of *B. rapa* (AA, 2n = 2x = 20). Both *B. rapa* and *A. thaliana* belong to the cruciferae family. *B. rapa* has undergone additional whole genome triplication (WGT) since its divergence from *A. thaliana*, followed by extensive gene loss and divergence of three subgenomes (Wang et al., 2011; Cheng et al., 2012a). Although the leaf ad-ab patterning mechanism in *A. thaliana* has been extensively studied, that in Chinese cabbage and its association with head leaf incurvature have not been investigated. The present study has identified genes correlated to leaf ad-ab polarity establishment in Chinese cabbage by conducting comparative genomic analysis of *B. rapa* and *A. thaliana*, as well as analyzed the retention of all ad-ab patterning genes in *B. rapa* after WGT. We investigated the genetic variation in 45 leaf ad-ab patterning genes in a 94-accession collection, including heading and non-heading morphotypes of *B. rapa*. We further assessed their footprints of selection by using Tajima's D index and nucleotide diversity (π) among various heading and non-heading accessions. The results of the present study may contribute to the establishment of the genetic mechanism underlying leafy head formation in Chinese cabbage.

## Materials and methods

### Database for Ad-Ab patterning genes in *B. rapa*

The sequences of *A. thaliana* genes that are involved in the ad-ab patterning pathway were downloaded from the TAIR database (http://www.arabidopsis.org/). The *B. rapa* genome (version 1.5) and gene sequences from BRAD (http://brassicadb.org/brad/) (Cheng et al., 2011) were used to identify the ad-ab patterning genes in *B. rapa.*

### Identification of homologous genes between *B. rapa* and *A. thaliana*

The ad-ab patterning gene DNA and protein sequences of *A. thaliana* and *B. rapa* were respectively aligned using BLASTN and BLASTP using a cut off E-value of ≤1E-10 and coverage of ≥0.75. Syntenic orthologs between *A. thaliana* and *B. rapa* from BRAD (http://brassicadb.org/brad/) were determined based on sequence similarity (cutoff: E ≤ 10-20) and collinearity of flanking genes (Cheng et al., 2012b).

### Selection of accessions

To represent the maximum morphotype diversity of *B. rapa*, a total of 94 *B. rapa* accessions were selected from a larger collection that was used in a resequencing project involving SNP genotyping and phenotyping (Table S3). The selected accessions were also classified into two groups based on their phenotypes: 47 heading *B. rapa* (H-Br) and 47 non-heading *B. rapa* (NH-Br). The phylogenetic relationship of the 94 accessions has been presented in Zhao et al. (2005). Another 150 H-Br and 150 NH-Br accessions were further collected to confirm the purifying selection signal of *BrARF3.1*.

### π assessment and neutrality testing

By using resequencing and genotyping data from the 94 accessions, genetic diversity indices were calculated. Using the Variscan software (Hutter et al., 2006), neutrality (Tajima's D) (Tajima, 1989) was initially estimated for the entire genome by 10-kb sliding window analysis of all three groups (All, NH-Br, and H-Br), with test statistic values at the negative and positive 5 percentiles of the genome-wide distribution set as thresholds. Then, the π and Tajima's D index of the coding regions of the 45 candidate genes were estimated in all three groups using Variscan.

### Availability of supporting data

All the 26 genes of *A. thaliana* referred in this report were retrieved from the TAIR database.

The *B. rapa* genome sequence (version 1.5), as well as the coding and protein sequences of the 45 ad-ab patterning genes were acquired from the *Brassica* database (BRAD).

## Results

### Identification of leaf Ad-Ab patterning genes in *B. rapa*

A total of 26 genes involved in leaf ad-ab patterning have been identified in *A. thaliana*, which include 17 genes encoding for seven TF families of leaf polarity determinants, namely, 6 adaxial determinants, 6 middle domain determinants, and 5 abaxial determinants; the remaining 9 genes are involved in the production and activity of small RNA determinants (miR165/166 and ta-siRNA) (Table 1). By using a combination of syntenic and non-syntenic homology analyses (Guo et al., 2014) and the 26 homologs in *A. thaliana*, we identified 45 leaf ad-ab patterning genes in the draft genome of *B. rapa* (v1.5), which consists of a total of 41,020 annotated genes (Wang et al., 2011). Thirty-two of the 45 genes were TF determinants, whereas 13 were responsible for small RNA pathway determinants. Each gene in *B. rapa* was assigned a name based on its *A. thaliana* homolog.

**Table 1:** **Ad-ab patterning genes identified in *Brassica rapa***.

**Gene family**	**Gene**	***Arabidopsis thaliana* gene**	***Brassica rapa*^a^**	**Gene**	**Subgenome^b^**	**Block^c^**	**Chromosome**
**TRANSCRIPTIONAL FACTORS REGULATORS OF LEAF POLARITY**							
**Adaxial determinants**							
HD-ZIP III	*REV*	AT5G60690	*BrREV.1*	(Bra002458)	LF	Wb	A10
			*BrREV.2*	(Bra020236)	MF2	Wb	A02
			BrREV.3^*^	(Bra038295)	LF	M	A06
	*PHB*	AT2G34710	*BrPHB.1*	(Bra005398)	LF	J	A05
			*BrPHB.2*	(Bra021926)	MF1	J	A04
	*PHV*	AT1G30490	*BrPHV*	(Bra032394)	LF	B	A09
	*ATHB8*	AT4G32880	*BrHB8.1*	(Bra011392)	LF	U	A01
			*BrHB8.2*	(Bra034539)	MF2	U	A08
ARP	*AS1*	AT2G37630	*BrAS1.1*	(Bra005177)	LF	J	A05
			*BrAS1.2*	(Bra000011)	MF2	J	A03
LOB	*AS2*	AT1G65620	*BrAS2*	(Bra039733)	MF1	E	A02
**Middle domain determinants**							
YABBY	*YAB1*	AT2G45190	*BrYAB1.1*	(Bra040322)	MF1	J	A04
			*BrYAB1.2*	(Bra000378)	MF2	J	A03
			BrYAB1.3^*^	(Bra003309)	MF2	N	A07
	*YAB2*	AT1G08465	*BrYAB2.1*	(Bra018624)	LF	A	A06
			*BrYAB2.2*	(Bra030728)	MF1	A	A08
			*BrYAB2.3*	(Bra031629)	MF2	A	A09
	*YAB3*	AT4G00180	*BrYAB3*	(Bra037320)	LF	O	A09
	*YAB5*	AT2G26580	*BrYAB5*	(Bra000538)	MF2	I	A03
WOX	*WOX1*	AT3G18010	*BrWOX1.1*	(Bra022267)	LF	F	A05
			*BrWOX1.2*	(Bra001694)	MF2	F	A03
	*WOX3*	AT2G28610	*BrWOX3.1*	(Bra035688)	MF1	I	A04
			*BrWOX3.2*	(Bra000484)	MF2	I	A03
**Abaxial determinants**							
KANADI	*KAN1*	AT5G16560	*BrKAN1*	(Bra008613)	LF	R	A10
	*KAN2*	AT1G32240	*BrKAN2.1*	(Bra023254)	LF	B	A09
			*BrKAN2.2*	(Bra033844)	MF2	B	A05
	*KAN3*	AT4G17695	*BrKAN3.1*	(Bra040176)	LF	U	A01
			*BrKAN3.2*	(Bra021038)	MF2	U	A08
ARF	*ARF3*	AT2G33860	*BrARF3.1*	(Bra005465)	LF	J	A05
			*BrARF3.2*	(Bra021885)	MF1	J	A04
	*ARF4*	AT5G60450	*BrARF4.1*	(Bra002479)	LF	Wb	A10
			*BrARF4.2*	(Bra020243)	MF2	Wb	A02
**SMALL RNA PATHWAY COMPONENTS INVOLVED IN LEAF POLARITY**							
**Generators of small RNAs**							
AGO	*AGO1*	AT1G48410	*BrAGO1.1*	(Bra032254)	MF2	C	A05
			BrAGO1.2^*^	(Bra014136)	MF1	C	A08
	*AGO7*	AT1G69440	*BrAGO7*	(Bra003999)	MF2	E	A07
	*AGO10*	AT5G43810	*BrAGO10.1*	(Bra033698)	LF	V	A06
			*BrAGO10.2*	(Bra027505)	MF2	V	A09
SGS	*SGS3*	AT5G23570	*BrSGS*	(Bra009695)	LF	Q	A06
RDR	*RDR6*	AT3G49500	*BrRDR6*	(Bra029957)	MF1	M	A01
HYL	*HYL1*	AT1G09700	*BrHYL1.1*	(Bra019999)	LF	A	A06
			*BrHYL1.2*	(Bra030793)	MF1	A	A08
DCL	*DCL1*	AT1G01040	*BrDCL1*	(Bra033293)	LF	A	A10
	*DCL4*	AT5G20320	*BrDCL4*	(Bra002293)	LF	R	A10
SE	*SE*	AT2G27100	*BrSE.1*	(Bra012034)	LF	I	A07
			*BrSE.2*	(Bra034322)	MF1	I	A04

a Asterisks indicate genes that do not exhibit synteny.

b The three subgenomes include the least fractionated blocks (LF), the medium fractionated blocks (MF1), and the most fractionated blocks (MF2).

c Characters referring to 24 conserved blocks (A–X) represent the conserved segments identifiable in the ancestral karyotype, A. thaliana, and B. rapa (Schranz et al., 2006).

Comparative analysis of syntenic orthologs of *A. thaliana* and *B. rapa* showed that 42 of the 45 genes were syntenic to 26 genes in *A. thaliana*. No tandem clusters involving these genes were identified. Wang et al. (2011) inferred three subgenomes in *B. rapa*, namely, the least fractionated blocks (LF), the medium fractionated blocks (MF1), and the most fractionated blocks (MF2). Based on this subgenome information, 21, 9, and 15 genes were distributed across LF, MF1, and MF2, respectively. Of the 42 syntenic orthologs, 20 were on LF, 8 were on MF1, and 14 were on MF2. The number of syntenic orthologs distributed across LF was more than those in MF1 and MF2, respectively, indicating that the subgenomic distribution of the ad-ab patterning genes is consistent with the gene fractionation status at the whole-genome level.

### Expansion of leaf Ad-Ab patterning genes in *B. rapa*

Only 62% of the *A. thaliana* orthologs harbored more than one paralogous copy in *B. rapa*, which was suggestive of the deletion of one or more paralogs via diploidization after WGT. To further observe the retention status of the leaf ad-ab patterning genes after WGT of the *B. rapa* genome, we investigated the number and ratio of single-copy to multiple-copy paralogous genes. We also used the ratio of the total number of single- to multiple-copy (two and three) paralogous genes (genes in the same tandem array were only counted once) of the entire *B. rapa* genome as background (1.342; 9175/6836) (Li et al., 2014) to calculate the *P*-value using Fisher's t-test. The *P*-value of the TF pathway ad-ab patterning genes (0.42; 5/12) was 0.026, indicating that these were significantly lower than that of the background, whereas no differences between the small RNA pathway ad-ab patterning genes (1.25; 5/4) and total ad-ab patterning genes (0.63; 10/16) and the background were observed (*P* > 0.05; Table 2). These results were suggestive of over-retention of TF pathway ad-ab patterning genes, but not total or small RNA pathway ad-ab patterning genes in *B. rapa*.

**Table 2:** **Number and ratio of single-copy to multiple-copy paralogs of *Arabidopsis* ad-ab patterning genes**.

	**Copy number variations^a^**				**Ratio of single to^b^**	***P*-value^c^**
	**One**	**Two**	**Three**	**Total**	**Multiple copies**	
TF pathwa y genes	5	9	3	17	5:12	0.02595^*^
Small RNA pathway genes	5	4	0	9	5:4	1
Total	10	13	3	26	10:16	0.07229

a Number of A. thaliana paralogs with different numbers of syntenic copies in B. rapa.

b The ratio of single to multiple copies is the number of paralogs having one copy vs. the total number of paralogs with two and three copies.

c The proportion of total paralogous sets with different copy numbers over the entire genome was used as background to calculate the P-value using Fisher's test.

* P < 0.05.

### Polymorphisms in *B. rapa* leaf Ad-Ab patterning genes

We estimated the genetic variation of 45 ad-ab patterning genes by examining our resequencing data set, which consisted of 94 double haploid (DH) accessions that represented different geographic origins and all known *B. rapa* morphotypes selected from a larger sample collection used in a whole-genome resequencing project (data not published). We called out all sequence variations within these 45 genes (including exons and introns). A total of 953 SNPs were detected. A total of 539 SNPs were identified within coding sequences (CDS), which included 419 synonymous and 120 non-synonymous SNPs. Details on the distribution of SNPs are presented in Table 3.

**Table 3:** **Genetic diversity in ad-ab patterning genes in 94 accessions of *B. rapa***.

**Gene**	**Number of exons**	**CDS length**	**Total number of SNPs**	**Number of SNPs in CDS**	**No. of non-syn SNPs**	**No. of syn SNPs**
*BrREV.1*	17	2553	67	41	1	40
*BrREV.2*	17	2541	25	18	6	12
*BrREV.3*	18	2502	11	5	2	3
*BrPHB.1*	18	2550	37	18	4	14
*BrPHB.2*	15	2532	20	11	1	10
*BrPHV*	17	2523	15	6	2	4
*BrHB8.1*	16	2502	1	0	0	0
*BrHB8.2*	17	2499	3	0	0	0
*BrAS1.1*	1	1086	4	4	1	3
*BrAS1.2*	1	1044	7	7	1	6
*BrAS2*	1	609	2	2	0	2
*BrKAN1*	4	876	26	8	2	6
*BrKAN2.1*	6	1101	14	2	0	2
*BrKAN2.2*	6	1179	13	2	1	1
*BrKAN3.1*	6	954	31	11	5	6
*BrKAN3.2*	5	540	8	2	2	0
*BrYAB1.1*	7	702	4	1	0	1
*BrYAB1.2*	7	678	2	1	0	1
*BrYAB1.3*	6	633	3	0	0	0
*BrYAB2.1*	5	513	8	2	0	2
*BrYAB2.2*	5	474	2	0	0	0
*BrYAB2.3*	6	564	2	0	0	0
*BrYAB3*	7	717	0	0	0	0
*BrYAB5*	6	495	6	2	0	2
*BrARF3.1*	9	1818	11	10	3	7
*BrARF3.2*	9	1659	26	18	7	11
*BrARF4.1*	12	2277	7	6	2	4
*BrARF4.2*	3	435	10	8	5	3
*BrWOX1.1*	4	1056	23	11	8	3
*BrWOX1.2*	4	1062	30	15	8	7
*BrWOX3.1*	2	705	0	0	0	0
*BrWOX3.2*	2	681	2	2	2	0
*BrAGO10.1*	17	2925	38	21	1	20
*BrAGO10.2*	4	423	3	2	2	0
*BrAGO1.1*	21	3336	51	13	3	10
*BrAGO1.2*	21	3240	91	47	1	46
*BrAGO7*	3	2946	40	38	7	31
*BrSGS*	5	1821	18	15	2	13
*BrRDR6*	2	3597	32	29	7	22
*BrHYL1.1*	3	825	9	6	1	5
*BrHYL1.2*	3	837	11	2	0	2
*BrDCL1*	18	5541	94	76	12	64
*BrDCL4*	25	4926	64	27	9	18
*BrSE.1*	12	2133	41	20	5	15
*BrSE.2*	11	2079	41	30	7	23

Syn, synonymous substitutions; Non-syn, non-synonymous substitutions; CDS (Coding sequence, only exons); gene (including exons and introns).

Nucleotide diversity (π) significantly varied among the 45 candidate genes. The genes encoding for TF pathway determinants exhibited a lower π than those involved in small RNA pathway determinants (Table S1). For the TF pathway genes, the YABBY gene family showed the lowest number of nucleotide substitutions (π range: 0.000233–0.001081), followed by the KAN family genes (π range: 0.000957–0.001993), whereas the WOX gene family showed higher variability (π range: 0.000924–0.00628). By comparison, the genes involved in small RNA pathways showed a higher number of polymorphisms (π range: 0.001704–0.007578). More specifically, two genes (*BrWOX3.1* and *BrYAB3*) show no variations in gene regions, whereas 14 genes (*BrAS2, BrHB8.1, BrHB8.2, BrHYL1.2, BrKAN2.1, BrWOX3.1*, and all the eight YABBY family genes) did not harbor any non-synonymous mutations within coding regions.

**Figure 2:**
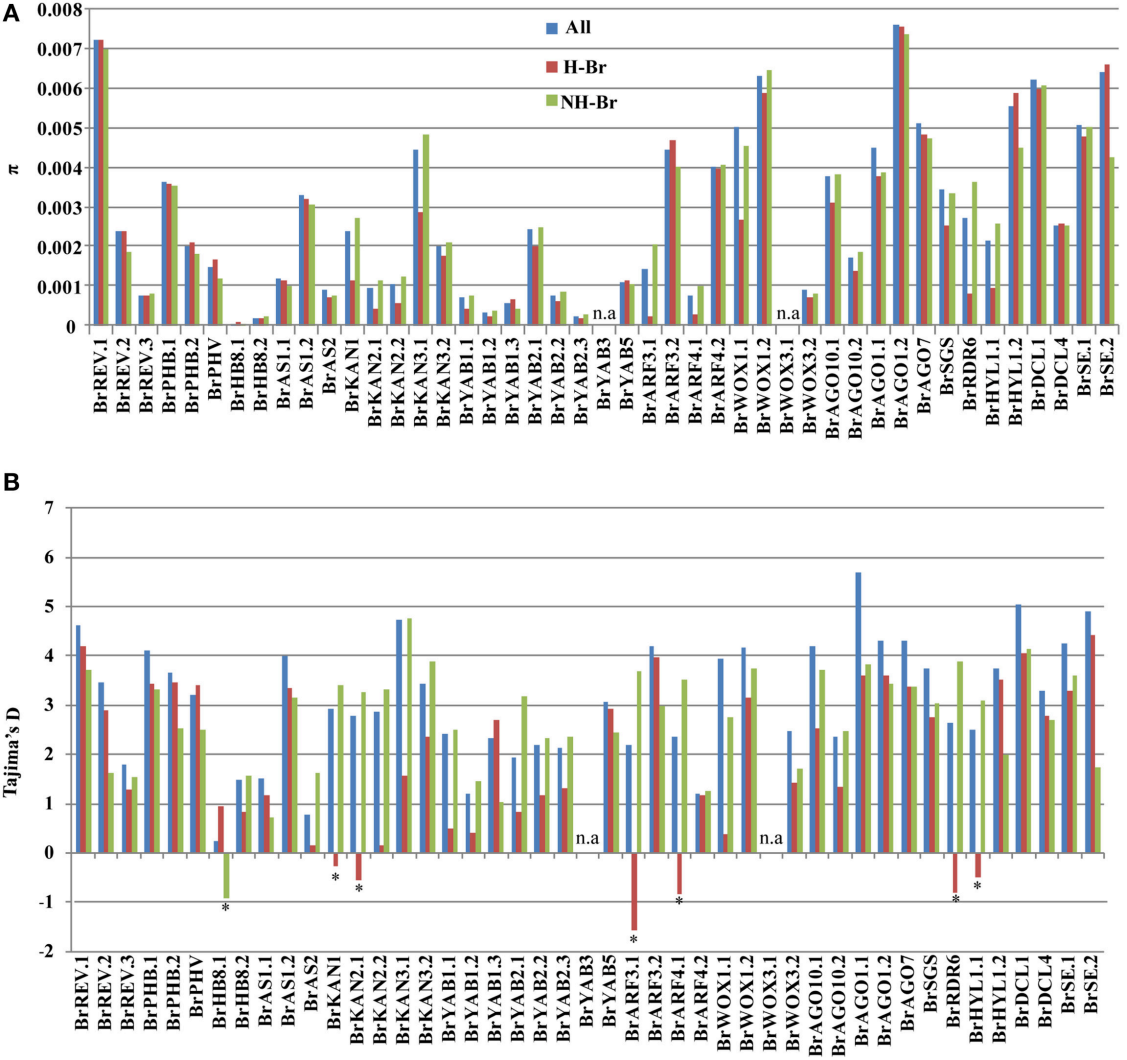
**Nucleotide diversity (π) (A) and Tajima's D (B) indices in 45 candidate genes of three groups: whole collection (All), and H-Br and NH-Br groups**. The genes for which Tajima's D is significant in the 94 accessions collection are indicated (^*^P < 0.05), as well as in All, H-Br, and NH-Br subgroups.

To detect the correlation between the pattern of nucleotide diversity among leaf ad-ab pattering genes and the leaf morphotype in *B. rapa*, nucleotide variation and diversity of the 45 candidate genes were separately estimated by grouping the 94 accessions into two different morphotype classes: 47 were Chinese cabbage as heading *B. rapa* (H-Br), whereas the other 47 were non-heading *B. rapa* (NH-Br). Approximately 10 genes *(BrARF3.1, BrARF4.1, BrKAN1, BrKAN2.1, BrKAN2.2, BrKAN3.1, BrHYL1.1, BrRDR6, BrYAB1.1*, and *BrWOX1.1*) exhibited lower π in H-Br compared to those of NH-Br, whereas *BrSE.2* and *BrHYL1.2* showed lower π in NH-Br than that observed in H-Br (Figure 2A). However, the remaining 33 genes in the NH-Br and H-Br groups did not exhibit substantial differences in diversity.

### Inferred selection patterns of the Ad-Ab patterning genes across various genetic groups

To determine whether the reduction in nucleotide diversity of the candidate genes were caused by artificial selection, Tajima's D test was used to determine the departure from neutrality of the 45 candidate gene regions in all three groups (All, HBr, and NH-Br). Neutrality was first estimated for the entire genome using sliding window analysis of all three groups. Figure S1 shows that these distributions substantially deviated in all three groups. Based on this finding, we set the test statistic values at the negative and positive 5 percentiles of the genome-wide distribution as thresholds. Neutrality test statistics values for the 45 candidate genes were considered significant when these exceeded the threshold value (Table S2).

Six of the 45 genes underwent negative selection in the H-Br group. All 44 candidate genes, except for *BrAGO1.1*, did not show any significant negative or positive Tajima's D value in the entire collection (Table S1), whereas *BrAGO1.1* showed a significant positive Tajima's D value. Although 10 genes showed significantly reduced π in the H-Br group compared to that in the NH-Br group (Figure 2A), six of these (*BrARF3.1, BrARF4.1, BrKAN1, BrKAN2.1, BrRDR6*, and *BrHYL1.1*) yielded significant negative Tajima's D indices in the H-Br group. On the other hand, positive yet not significant indices were obtained in the NH-Br group (Figure 2B). These findings suggest that purifying selection had acted on the six genes in the H-Br group. In addition, one gene (*BrHB8.1*) showed a significant negative Tajima's D value, although no differences in π between the NH-Br and H-Br groups were observed. The remaining 38 genes showed positive Tajima's D values between the H-Br and NH-Br groups, although not statistically significant (Figure 2B), indicating that these did not significantly differ from neutral expectations. Combined π analysis and neutrality testing suggested that 6 of the 45 genes (*BrARF3.1, BrARF4.1, BrKAN1, BrKAN2.1, BrRDR6*, and *BrHYL1.1*) probably underwent purifying selection in the H-Br group.

**Figure 3:**
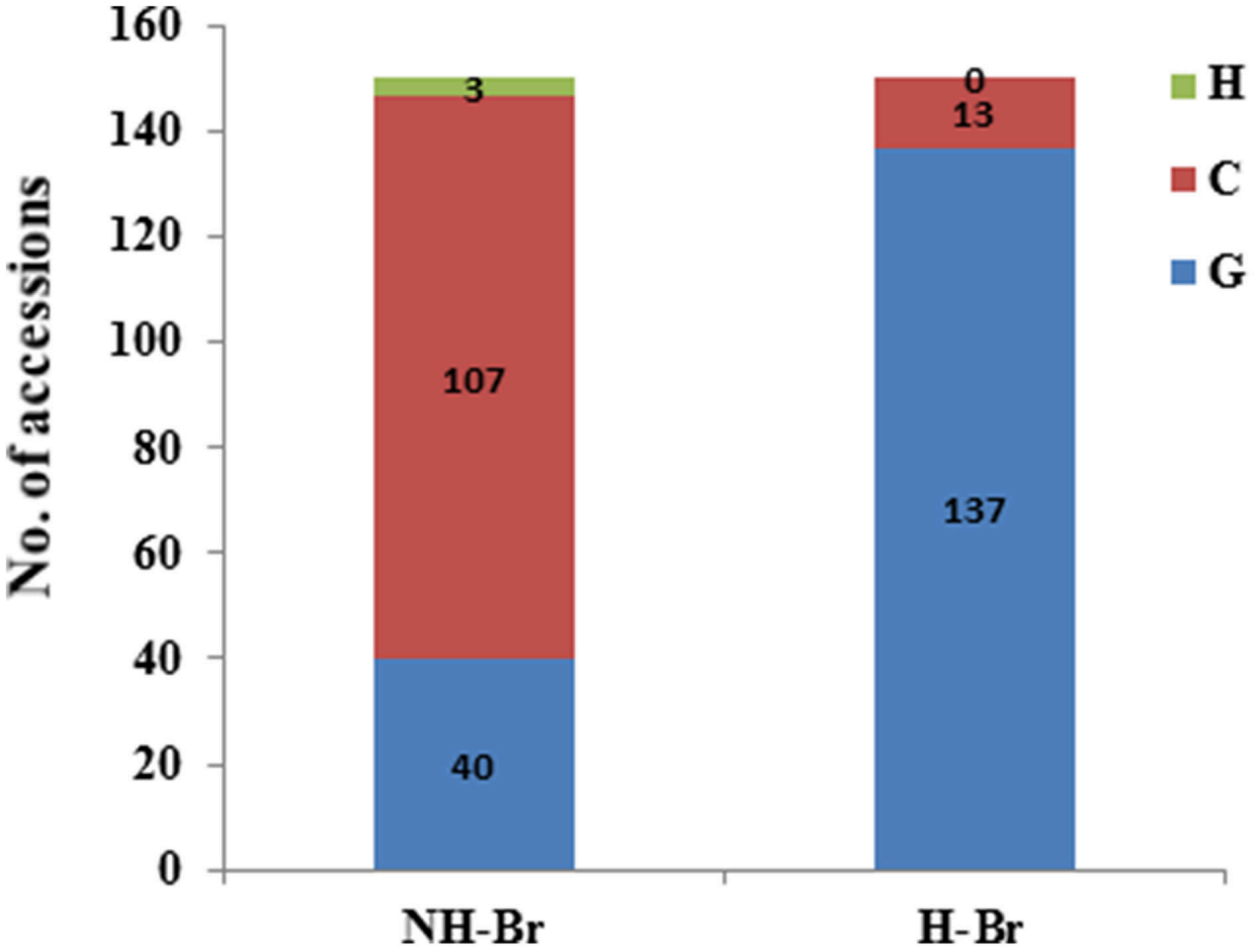
**Nucleotide bias distribution in the *BrARF3.1* gene between heading and non-heading *B. rapa* accessions**. A non-synonymous mutation (G to C) in the *BrARF3.1* gene was observed in a larger *B. rapa* germplasm collection consisting of 300 accessions; C, G, and H represent the genotype of C, G and hybrid, respectively.

To verify the reliability of the selection signals, we further examined *BrARF3.1* using an expanded collection consisting of 300 accessions (Table S4). Three non-synonymous substitutions were detected in the coding region of *BrARF3.1* (Table S5). The A/G SNP at position 568 and the T/C SNP at position 1299 was predicted to result in an amino acid change of Val/Ile and Val/Ala, respectively, but affected neither the charge of the amino acid R-chain nor the polarity of the amino acid. The G/C SNP in exon 9 at gene position 2263 (CDS position: 1563 bp) was predicted to result in an amino acid change from an uncharged Gln to a positively charged His (Figure S2), which results in the alteration of protein function, as well as a significant difference between the H-Br and NH-Br groups (*P* = 2.25E-9) by using the Fisher's test (Table 4). We then designed a CAPS marker to genotype the G/C mutation in an extended germplasm collection consisting of 300 accessions, which included 150 heading and 150 non-heading cultivars (Table S3). Our results showed that 91.3% of the heading accessions were of the G genotype, and only 8.7% of the accessions were of the C genotype. On the other hand, in the non-heading group, 71.3% of the accessions were of the C genotype, whereas 26% were of the G genotype, and Fisher's exact test showed a *P*-value of 6.93E-32 (Figure 3). These results confirmed that this substitution clearly differentiated H-Br from NH-Br, thus validating our hypothesis that the *BrARF3.1* gene in the H-Br group underwent purifying selection.

**Table 4:** **Characteristics and prediction of non-synonymous changes in the *BrARF3.1* gene**.

**Genome position (bp)**	**SNP**	**Amino acid change**	**Amino acid substitution characteristics**	***P*-value^a^**
5,653,931	SNP 568 (A/G)	V127I	Non-polar neutral	7.66E-07
5,654,662	SNP 1299 (T/C)	V284A	Non-polar neutral	0.000268
5,655,627	SNP 2263 (G/C)	Q521 H	Non-polar neutral/basic polar positive	2.25E-09

Sites are identified based on the reference locus BrARF3.1.

a Substitutions between H-Br and NH-Br were estimated with the P-value using Fisher's test.

### Differentiation of leaf Ad-Ab patterning genes in *B. rapa*

*B. rapa* underwent WGT after its divergence from *A. thaliana*. Whole-genome comparative analysis using 26 homologs in *A. thaliana* identified 45 ad-ab patterning genes in *B. rapa*, which suggested that the ad-ab patterning genes in *B. rapa* underwent duplication after the WGT event. The sequences of these 45 genes exhibited significant variation relative to the 94 *B. rapa* collections. Moreover, a higher level of sequence variation was observed among the paralogous copies. The KAN and ARF gene families are key regulators of abaxial polarity in leaves, and the absence of *KAN* or *ARF* function resulted in leaf curvature in *A. thaliana*, similar to that of Chinese cabbage. Taking *BrKAN* and *BrARF* genes as examples, five *BrKAN* genes as homologs of three *AtKAN* genes exhibited significant differences in nucleotide diversities within all 94 collections (Table S2, Figure 2). The highest diversity was observed in *BrKAN3.1*. Nucleotide diversity within the *BrKAN3.1* gene was 10-fold higher than that observed in *BrKAN2.1* and *BrKAN2.2*. Moreover, all the *BrKAN* genes, instead of only the *BrKAN3.2* gene, showed lower π values in the H-Br group compared to that in the NH-Br group. However, only *BrKAN1* and *BrKAN2.1* showed significant negative Tajima D values, whereas the other *BrKAN* genes showed positive Tajima D values, although not statistically significant. The ARF gene family also showed different π values and selection patterns. The *BrARF3.1* gene showed a lower degree of π than *BrARF3.2*, and comparison of these two paralogous genes indicated that the *BrARF3.1* gene had reduced π and a significant negative Tajima's D value in the H-Br group, but not in the NH-Br group. On the other hand, no differences in π and Tajima's D value for the *BrARF3.2* gene were observed between the H-Br and NH-Br groups. Similar findings were obtained from the paralogous pair *BrARF4.1* and *BrARF4.2*. These results suggested that each of the *BrKAN* and *BrARF* orthologous genes has a different number of duplicated copies, which in turn results in variable π values and selection patterns. In general, duplications are assumed to be essential to the generation of novel gene functions, as well as to the alterations in patterns of expression (Lynch and Conery, 2000). However, several duplicated genes may have experienced relaxed selection, but when duplicates acquire different functions, these are potentially exposed to gradually increasing selective constraints (Lynch and Conery, 2000). In the present study, the duplicated paralogous genes might have undergone functional divergence during the leaf ad-ab patterning pathway in *B. rapa.*

## Discussion

### Leaf Ad-Ab patterning and leaf morphology diversity

Angiosperm leaves show remarkable morphological diversity, and evolutionary studies on various leaf forms such as unifacial, peltate, and compound leaves have suggested that alterations in the ad-ab patterning mechanism causes dramatic modifications in leaf morphology and thus, could be a major driving force in the generation of diverse leaf forms (see review Yamaguchi et al., 2012). *B. rapa* comprises a variety of vegetables with rich leaf morphological diversity. In Chinese cabbage, leaf morphology is highly variable, which include seedling leaf, rosette leaf, and head leaf. Seedling leaves and rosette leaves are flat and function in photosynthesis and respiration, whereas head leaves show incurvature and are responsible for the formation of a leafy head. In the present study, the observed diversity in leaf morphology in Chinese cabbage might have been caused by alterations in leaf ad-ab patterning. In addition, previous studies on leaf ad-ab patterning mutants present the leaf curling phenotype (Yamaguchi et al., 2012), and leaf incurvature is essential for leafy head formation (Mao et al., 2014), further indicating that leaf ad-ab patterning could be involved in head leaf incurvature and subsequent leafy head formation. However, studies on leaf ad-ab patterning in *B. rapa* are limited. The aim of the present study was to identify the ad-ab patterning genes in *B. rapa* and to investigate whether these were involved in leafy head formation in Chinese cabbage.

### Six leaf Ad-Ab patterning genes are under selection in chinese cabbage

Comparative analysis using *A. thaliana* has identified 45 ad-ab patterning genes in *B. rapa*. Our establishment of a set of ad-ab patterning genes in *B. rapa* will not only provide a valuable resource for the study of the leaf ad-ab patterning system of *B. rapa*, but also will facilitate in elucidating its role in leafy head formation in Chinese cabbage.

We also assessed intraspecific nucleotide and modes of selection for the 45 ad-ab polarity genes in a collection of 94 accessions that included the heading and non-heading morphotype. All 45 genes exhibited significant sequence variation; however, these did not follow any common pattern and was strongly dependent on its gene family. Moreover, members of the same gene family or even paralogous copies exhibited significant variation. However, most of the genes exhibited similar π values and did not show significant selection signals between the NH-Br and H-Br groups. Only six genes (*BrARF3.1, BrARF4.1, BrKAN1, BrKAN2.1, BrRDR6*, and *BrHYL1.1*) showed significant negative Tajima's D values that were in agreement with the observed reduction in π value in the H-Br group, which was suggestive of purifying selection. However, by using these analytical methods, we could not avoid acquiring false positive selection or false negative signals. Such reduced diversity could also be explained by background selection, genetic hitchhiking, or by an extension of the effective population size following a bottleneck. Despite these limitations, the present study highlighted that purifying selection of these six genes in the H-Br group could not be ruled out, and the selection signal of the *BrARF3.1* gene was subsequently confirmed in a larger group of accessions. We thus postulated that these six genes could serve as candidate genes that are responsible for leafy head formation in Chinese cabbage and its functional characterization should be investigated in future studies.

### Leaf Ad-Ab patterning genes are probably linked to leafy head formation in chinese cabbage

The present study has determined that 6 of the 45 ad-ab patterning genes in the H-Br group underwent strong negative selection. Of these six genes, *BrARF3.1, BrARF4.1, BrKAN1*, and *BrKAN2.1* were identified as homologs of *Arabidopsis* abaxial polarity genes, and *BrHYL1.1* is the homolog of *Arabidopsis HYL*, which is involved in the biogenesis of the abaxial determinant, miRNA166. On the other hand, the *RDR6* gene encodes for small RNA determinants, including adaxial and abaxial determinants. These findings indicated that all six genes, except for *BrRDR6*, were involved in leaf abaxial polarity. Previous studies on *Arabidopsis* have suggested that ARF3 and ARF4 act together with KANs in the determination of leaf abaxial fate (Pekker et al., 2005). Individual loss-of-function mutants of *ARF* (*ARF3* and *ARF4*) or *KAN* (*KAN1–KAN3*) show weak or no phenotypes in leaves; however, double mutants of *arf3arf4* develop leaves that are curled up, resembling the phenotype of *kan1 kan2* leaves (Eshed et al., 2001, 2004; Pekker et al., 2005), thereby indicating an overlap in the function of leaf abaxial polarity. As expected, the homologous genes of *ARF3, ARF4, KAN1*, and *KAN2* in *B. rapa* were all included in the candidate gene list for leaf incurvature formation. Therefore, our results suggest that leaf ad-ab patterning is associated to leaf incurvature within the leafy head of Chinese cabbage. This hypothesis is supported by the evidence that *BcpLH* (also known as *BrHYL*) plays a role in the inward orientation of head leaves of Chinese cabbage.

Taken together, our present study has provided a set of candidate genes that are responsible for leafy head formation in Chinese cabbage. Functional analysis of these candidate genes and anatomy analysis of ad-ab patterning in heading leaves compared to those of flat rosette leaves may improve our understanding of the mechanism underlying leafy head formation.

## Author contributions

XW and FC designed the research. JL and BL performed the research and analyzed the data. FC and JW contributed computational tools and data. JL wrote the article. XW, JW and FC reviewed the manuscript.

### Conflict of interest statement

The authors declare that the research was conducted in the absence of any commercial or financial relationships that could be construed as a potential conflict of interest. The reviewer Stefan De Folter and Handling Editor declared their shared affiliation, and the Handling Editor states that the process nevertheless met the standards of a fair and objective review.
