# Supplementary material for: Genetic Variation and Divergence of Genes Involved in Leaf Adaxial-Abaxial Polarity Establishment in Brassica rapa
Source: Front Plant Sci. 2016 Feb 9;7:94. doi: 10.3389/fpls.2016.00094 (PMC4746309; doi:10.3389/fpls.2016.00094)
Supplement: Supplementary file 1 [file DataSheet1.PDF]

Table S1. List of nucleotide diversity (π) and Tajima' D for each candidate according to genetic groups: whole collection(All), H-Br and NH-Br groups

Gene	π			TajimaD / Sig D					
	All	H-Br	NH-Br	All		H-Br		NH-Br	
BrREV.1	0.007201	0.007225	0.0069757	4.6182389	n.s.	4.184139	n.s.	3.722391	n.s.
BrREV.2	0.002366	0.00236	0.0018529	3.4550427	n.s.	2.908439	n.s.	1.637328	n.s.
BrREV.3	0.000775	0.000749	0.0008021	1.7834272	n.s.	1.270964	n.s.	1.546125	n.s.
BrPHB.1	0.003615	0.003569	0.0035151	4.1195985	n.s.	3.431887	n.s.	3.332889	n.s.
BrPHB.2	0.001984	0.002093	0.0017864	3.6546708	n.s.	3.468768	n.s.	2.533305	n.s.
BrPHV	0.001469	0.001654	0.0011786	3.1958976	n.s.	3.417465	n.s.	2.489581	n.s.
BrHB8.1	4.82E-05	8.01E-05	0.0000095	0.2471421	*	0.948988	n.s.	-0.9111	**
BrHB8.2	0.000206	0.000179	0.0002276	1.4696773	n.s.	0.837074	n.s.	1.556284	n.s.
BrAS1.1	0.001165	0.001139	0.0009794	1.5029591	n.s.	1.165227	n.s.	0.721876	*
BrAS1.2	0.003308	0.003171	0.0030549	3.9981552	n.s.	3.358595	n.s.	3.149596	n.s.
BrAS2	0.000883	0.000702	0.0007664	0.775463	*	0.14466	n.s.	1.610845	n.s.
BrKAN1	0.002384	0.001148	0.0027338	2.9345872	n.s.	-0.28232	*	3.395573	n.s.
BrKAN2.1	0.000957	0.000416	0.0011325	2.7755566	n.s.	-0.54559	*	3.265525	n.s.
BrKAN2.2	0.001035	0.000577	0.0012152	2.8698264	n.s.	0.151649	n.s.	3.326518	n.s.
BrKAN3.1	0.004422	0.002875	0.0048278	4.728364	n.s.	1.576466	n.s.	4.760583	n.s.
BrKAN3.2	0.001993	0.001767	0.0021163	3.4430169	n.s.	2.366028	n.s.	3.873259	n.s.
BrYAB1.1	0.00071	0.000427	0.0007713	2.417594	n.s.	0.486712	n.s.	2.49352	n.s.
BrYAB1.2	0.000307	0.000235	0.0003571	1.2136148	n.s.	0.417805	n.s.	1.440924	n.s.
BrYAB1.3	0.000584	0.000678	0.0004257	2.3261738	n.s.	2.705678	n.s.	1.022268	n.s.
BrYAB2.1	0.002414	0.001978	0.002464	1.9462345	n.s.	0.821482	n.s.	3.186369	n.s.
BrYAB2.2	0.000778	0.0006	0.0008561	2.1815574	n.s.	1.174264	n.s.	2.337727	n.s.
BrYAB2.3	0.000233	0.000191	0.0002596	2.1459432	n.s.	1.320834	n.s.	2.346162	n.s.
BrYAB3	n.a	n.a	n.a	n.a		n.a		n.a	
BrYAB5	0.001081	0.001124	0.0010211	3.0640489	n.s.	2.916469	n.s.	2.442506	n.s.
BrARF3.1	0.001438	0.00022	0.0020456	2.1953707	n.s.	-1.58167	**	3.674735	n.s.
BrARF3.2	0.004419	0.004654	0.0039882	4.1988996	n.s.	3.979249	n.s.	2.978636	n.s.
BrARF4.1	0.000737	0.000258	0.0009918	2.3525724	n.s.	-0.84037	*	3.513824	n.s.
BrARF4.2	0.004007	0.003959	0.004064	1.2012424	n.s.	1.15945	n.s.	1.256818	n.s.
BrWOX1.1	0.005023	0.00265	0.0045167	3.9543683	n.s.	0.393212	n.s.	2.763115	n.s.
BrWOX1.2	0.00628	0.005867	0.0064188	4.1782959	n.s.	3.154934	n.s.	3.740283	n.s.
BrWOX3.1	n.a	n.a	n.a	n.a	n.a	n.a		n.a	
BrWOX3.2	0.000924	0.000723	0.000786	2.470879	n.s.	1.437462	n.s.	1.696792	n.s.
BrAGO10.1	0.003766	0.003086	0.0038279	4.207889	n.s.	2.520751	n.s.	3.709106	n.s.
BrAGO10.2	0.001704	0.001371	0.0018628	2.3540027	n.s.	1.341107	n.s.	2.473766	n.s.
BrAGO1.1	0.004499	0.003747	0.0038656	5.6840169	*	3.603559	n.s.	3.819392	n.s.
BrAGO1.2	0.007578	0.007537	0.0073616	4.3120289	n.s.	3.607904	n.s.	3.446844	n.s.
BrAGO7	0.005079	0.004836	0.0047208	4.3159751	n.s.	3.369494	n.s.	3.366596	n.s.
BrSGS	0.00343	0.002502	0.003325	3.7500224	n.s.	2.742206	n.s.	3.050803	n.s.
BrRDR6	0.002707	0.000805	0.0036155	2.638806	n.s.	-0.79469	*	3.8779	n.s.
BrHYL1.1	0.002149	0.000941	0.0025906	2.5091782	n.s.	-0.48459	*	3.080873	n.s.

BrHYL1.2	0.00555	0.00585	0.004485	3.7303539	n.s.	3.518846	n.s.	1.997295	n.s.
BrDCL1	0.006194	0.005962	0.0060385	5.0516656	n.s.	4.050587	n.s.	4.145088	n.s.
BrDCL4	0.002539	0.002565	0.0025347	3.2992131	n.s.	2.774127	n.s.	2.702543	n.s.
BrSE.1	0.005041	0.004788	0.0050071	4.2560597	n.s.	3.298617	n.s.	3.593785	n.s.
BrSE.2	0.006409	0.006564	0.0042532	4.9076044	n.s.	4.426552	n.s.	1.750381	n.s.

Table S2. The Tajima's D neutrality test statistics values at the negative and positive 5 and 1 percentiles of the genome-wide distribution

	95%	99%
H-Br	-0.22 –4.7	-0.91–4.95
NH-Br	0 –4.63	-0.1–4.95
All	0–5.37	-0.09 –5.58

Table S3. Information of 94 resequenced *B. rapa* accessions

**NH-Br group**		**H-Br group**	
**Sample**	**Type**	**Sample**	**Type**
DF11QG001	Pak choi	DF11QG021	Chinese cabbage
DF11QG002	Pak choi	DF11QG022	Chinese cabbage
DF11QG003	Pak choi	DF11QG023	Chinese cabbage
DF11QG004	Pak choi	DF11QG024	Chinese cabbage
DF11QG005	Pak choi	DF11QG025	Chinese cabbage
DF11QG006	Pak choi	DF11QG026	Chinese cabbage
DF11QG007	Pak choi	DF11QG027	Chinese cabbage
DF11QG008	Pak choi	DF11QG028	Chinese cabbage
DF11QG009	Pak choi	DF11QG029	Chinese cabbage
DF11QG010	Pak choi	DF11QG030	Chinese cabbage
DF11QG011	Pak choi	DF11QG031	Chinese cabbage
DF11QG012	Pak choi	DF11QG032	Chinese cabbage
DF11QG013	Pak choi	DF11QG033	Chinese cabbage
DF11QG014	Pak choi	DF11QG034	Chinese cabbage
DF11QG015	Pak choi	DF11QG035	Chinese cabbage
DF11QG016	Pak choi	DF11QG036	Chinese cabbage
DF11QG017	Pak choi	DF11QG037	Chinese cabbage
DF11QG018	Pak choi	DF11QG038	Chinese cabbage
DF11QG019	Wutacai	DF11QG039	Chinese cabbage
DF11QG020	Wutacai	DF11QG040	Chinese cabbage
DF11QG066	Tai cai	DF11QG041	Chinese cabbage
DF11QG067	Tai cai	DF11QG042	Chinese cabbage
DF11QG068	Tai cai	DF11QG043	Chinese cabbage
DF11QG069	Tai cai	DF11QG044	Chinese cabbage
DF11QG070	Zi Caitai	DF11QG045	Chinese cabbage
DF11QG071	Zi Caitai	DF11QG046	Chinese cabbage

DF11QG072	Zi Caitai	DF11QG047	Chinese cabbage
DF11QG073	Cai xin	DF11QG048	Chinese cabbage
DF11QG074	Cai xin	DF11QG049	Chinese cabbage
DF11QG075	Cai xin	DF11QG050	Chinese cabbage
DF11QG076	Cai xin	DF11QG051	Chinese cabbage
DF11QG077	Wild	DF11QG052	Chinese cabbage
DF11QG078	Oil sarson	DF11QG053	Chinese cabbage
DF11QG079	Broccolleto	DF11QG054	Chinese cabbage
DF11QG080	Yellow Sarson	DF11QG055	Chinese cabbage
DF11QG081	Yellow Sarson	DF11QG056	Chinese cabbage
DF11QG082	Komatsuna	DF11QG058	Chinese cabbage
DF11QG083	Komatsuna	DF11QG059	Chinese cabbage
DF11QG084	Mizuna	DF11QG060	Chinese cabbage
DF11QG085	Mizuna	DF11QG061	Chinese cabbage
DF11QG086	Turnip	DF11QG062	Chinese cabbage
DF11QG087	Turnip	DF11QG063	Chinese cabbage
DF11QG088	Turnip	DF11QG064	Chinese cabbage
DF11QG089	Turnip	DF11QG065	Chinese cabbage
DF11QG092	Rapid cycling	DF11QG090	Chinese cabbage
DF11QG094	Turnip	DF11QG091	Chinese cabbage
DF11QG095	Tai cai	DF11QG093	Chinese cabbage

Table S4. Information of a larger collection with 300 *B. rapa* accessions

**NH-Br group**		**H-Br group**	
**Sample Index**	**Type**	**Sample Index**	**Type**
1	Chinese cabbage	2	Wild
3	Chinese cabbage	3	Wild
5	Chinese cabbage	4	Wild
7	Chinese cabbage	5	Wild
8	Chinese cabbage	6	Wild
9	Chinese cabbage	7	Wild
11	Chinese cabbage	8	Wild
13	Chinese cabbage	9	Wild
16	Chinese cabbage	12	Wild
19	Chinese cabbage	13	Wild
21	Chinese cabbage	30	Broccolleto
23	Chinese cabbage	31	Broccolleto
25	Chinese cabbage	32	Broccolleto
27	Chinese cabbage	33	Broccolleto
34	Chinese cabbage	34	Broccolleto
35	Chinese cabbage	35	Broccolleto
38	Chinese cabbage	36	Broccolleto
39	Chinese cabbage	37	Broccolleto
40	Chinese cabbage	38	Broccolleto
56	Chinese cabbage	39	Broccolleto

57	Chinese cabbage	41	Pak choi
58	Chinese cabbage	42	Pak choi
59	Chinese cabbage	43	Pak choi
76	Chinese cabbage	44	Pak choi
78	Chinese cabbage	45	Pak choi
80	Chinese cabbage	46	Pak choi
100	Chinese cabbage	47	Pak choi
101	Chinese cabbage	48	Pak choi
102	Chinese cabbage	49	Pak choi
104	Chinese cabbage	50	Pak choi
105	Chinese cabbage	97	Oils
111	Chinese cabbage	98	Oils
119	Chinese cabbage	99	Oils
121	Chinese cabbage	100	Oils
123	Chinese cabbage	103	Oils
126	Chinese cabbage	104	Oils
128	Chinese cabbage	105	Oils
130	Chinese cabbage	106	Oilw
133	Chinese cabbage	107	Oilw
134	Chinese cabbage	108	Oilw
136	Chinese cabbage	171	Turnip
137	Chinese cabbage	172	Turnip
138	Chinese cabbage	173	Turnip
141	Chinese cabbage	174	Turnip
143	Chinese cabbage	175	Turnip
144	Chinese cabbage	176	Turnip
145	Chinese cabbage	177	Turnip
146	Chinese cabbage	178	Turnip
148	Chinese cabbage	179	Turnip
150	Chinese cabbage	180	Turnip
151	Chinese cabbage	213	Wutacai
152	Chinese cabbage	214	Wutacai
155	Chinese cabbage	215	Wutacai
157	Chinese cabbage	216	Wutacai
169	Chinese cabbage	217	Komatsuna
172	Chinese cabbage	218	Mizuna
175	Chinese cabbage	219	Mizuna
177	Chinese cabbage	220	Mizuna
179	Chinese cabbage	221	Mizuna
182	Chinese cabbage	223	Turnip
183	Chinese cabbage	224	Turnip
186	Chinese cabbage	225	Turnip
191	Chinese cabbage	226	Turnip
192	Chinese cabbage	227	Turnip
194	Chinese cabbage	228	Turnip
199	Chinese cabbage	229	Turnip

201	Chinese cabbage	230	Turnip
222	Chinese cabbage	231	Turnip
228	Chinese cabbage	232	Turnip
230	Chinese cabbage	233	Turnip
232	Chinese cabbage	290	Broccolleto
233	Chinese cabbage	291	Broccolleto
234	Chinese cabbage	292	Broccolleto
252	Chinese cabbage	293	Broccolleto
256	Chinese cabbage	294	Zi Caitai
257	Chinese cabbage	295	Zi Caitai
258	Chinese cabbage	297	Zi Caitai
260	Chinese cabbage	298	Zi Caitai
262	Chinese cabbage	299	Zi Caitai
264	Chinese cabbage	300	Zi Caitai
292	Chinese cabbage	301	Mizuna
297	Chinese cabbage	302	Komatsuna
305	Chinese cabbage	303	Komatsuna
321	Chinese cabbage	304	Wutacai
326	Chinese cabbage	306	Oils
335	Chinese cabbage	307	Oils
343	Chinese cabbage	308	Oils
353	Chinese cabbage	309	Oils
366	Chinese cabbage	310	Oils
370	Chinese cabbage	311	Oils
373	Chinese cabbage	312	Oils
382	Chinese cabbage	313	Oils
385	Chinese cabbage	314	Oils
392	Chinese cabbage	315	Oils
398	Chinese cabbage	316	Oils
401	Chinese cabbage	317	Oils
403	Chinese cabbage	318	Oils
408	Chinese cabbage	319	Oils
409	Chinese cabbage	320	Oils
440	Chinese cabbage	321	Oils
457	Chinese cabbage	322	Oils
462	Chinese cabbage	323	Oils
488	Chinese cabbage	575	Pak choi
595	Chinese cabbage	576	Pak choi
596	Chinese cabbage	577	Pak choi
597	Chinese cabbage	578	Pak choi
598	Chinese cabbage	579	Pak choi
599	Chinese cabbage	580	Pak choi
600	Chinese cabbage	581	Pak choi
601	Chinese cabbage	582	Pak choi
602	Chinese cabbage	583	Pak choi
603	Chinese cabbage	584	Pak choi

604	Chinese cabbage	585	Pak choi
605	Chinese cabbage	586	Pak choi
606	Chinese cabbage	587	Pak choi
607	Chinese cabbage	588	Pak choi
608	Chinese cabbage	589	Pak choi
609	Chinese cabbage	590	Pak choi
610	Chinese cabbage	591	Pak choi
611	Chinese cabbage	592	Pak choi
612	Chinese cabbage	593	Wutacai
613	Chinese cabbage	594	Wutacai
614	Chinese cabbage	640	Tai cai
615	Chinese cabbage	641	Tai cai
616	Chinese cabbage	642	Tai cai
617	Chinese cabbage	643	Tai cai
618	Chinese cabbage	644	Zi Caitai
619	Chinese cabbage	645	Zi Caitai
620	Chinese cabbage	646	Zi Caitai
621	Chinese cabbage	647	Cai xin
622	Chinese cabbage	648	Cai xin
623	Chinese cabbage	649	Cai xin
624	Chinese cabbage	650	Cai xin
625	Chinese cabbage	651	Wild
626	Chinese cabbage	652	Oils
627	Chinese cabbage	653	Broccolleto
628	Chinese cabbage	654	Yellow Sarson
629	Chinese cabbage	655	Yellow Sarson
630	Chinese cabbage	656	Komatsuna
631	Chinese cabbage	657	Komatsuna
632	Chinese cabbage	658	Komatsuna
633	Chinese cabbage	659	Komatsuna
634	Chinese cabbage	660	Turnip
635	Chinese cabbage	661	Turnip
636	Chinese cabbage	662	Turnip
637	Chinese cabbage	663	Turnip
638	Chinese cabbage	664	Turnip
639	Chinese cabbage	666	Rapid cycling
665	Chinese cabbage	668	turnip
667	Chinese cabbage	669	Caitai

Table S5. The non-synonymous changes in the six genes with negative Tajima's D values

Gene	Chromsome	Site (bp)	SNP	Amino acid change
BrARF3.1 (Bra005465)	A05	5653931	GTT/ATT	Val/Ile
	A05	5654662	GTT/GCT	Val/Ala
	A05	5655627	CAG/CAC	Gln/His
BrARF4.1 (Bra002479)	A10	9366954	GGT/GAT	Gly/Asp
	A10	9367219	TCT/ACT	Ser/Thr
BrKAN1 (Bra008613)	A10	11896208	TCT/TGT	Ser/Cys
	A10	11896315	ATC/GTC	Ile/Val
BrRDR6(Bra029957)	A01	14715806	CAT/AAT	His/Asn
	A01	14715824	AAT/CAT	Asn/His
	A01	14716370	CAC/GAC	His/Asp
	A01	14716541	CAA/AAA	Gln/Lys
	A01	14717965	TGT/TTT	Cys/Phe
	A01	14718709	ATC/AGC	Ile/Ser
	A01	14718821	TCA/ACA	Ser/Thr
BrHYL1.1 (Bra019999)	A06	3336943	GAC/GCC	Asp/Ala

No non-synonymous changes were found in *BrKAN2.1* gene.

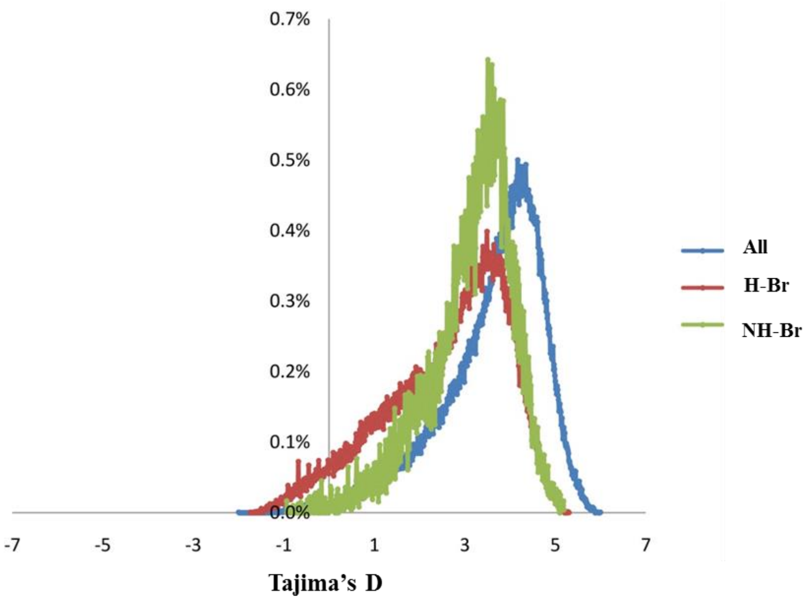

Figure S1. Estimated genome-wide distribution plots for Tajima's D neutrality test statistics. Blue lines represent genome-wide distribution on the whole collection, red and green lines represent the distribution on H-Br and NH-Br, respectively, based on the SNP from the resequencing data set. The *y* axis indicates the frequency of the test statistic value in the data set.

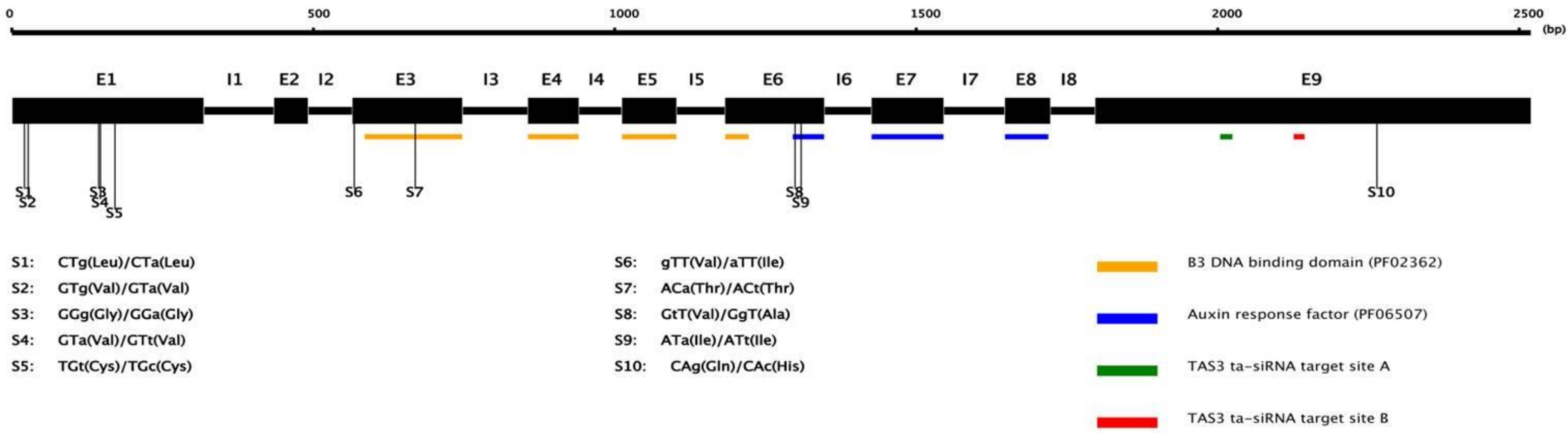

Figure S2. The non-synonymous and synonymous changes in *BrARF3.1*. Black blocks represent the exons, and black lines indicate the introns of *BrARF3.1*. S6, S8 and S10 are the non-synonymous changes, and the others are synonymous changes. Yellow blocks represent B3 DNA binding domains. Blue, green and red blocks indicate auxin response factor domain and TAS3 ta-siRNA target sites, respectively.
